# EVALUATION OF PHARMACY-BASED RECOMMENDATIONS WITHIN A COMPREHENSIVE GERIATRIC ASSESSMENT TEAM

**DOI:** 10.1093/geroni/igae098.2839

**Published:** 2024-12-31

**Authors:** Maria Cervantes, Nikil Patel, Jacob Tillmann, Christine Cigolle, Heather Bieber, Janette Dunlap, Kristin Phillips

**Affiliations:** Veterans Health Administration, St. Johns, Florida, United States; VA Ann Arbor Healthcare System, Ann Arbor, Michigan, United States; North Florida/South Georgia Veterans Health System, Gainesville, Florida, United States; VA Ann Arbor Healthcare System, Ann Arbor, Michigan, United States; North Florida/South Georgia Veterans Health System, Gainesville, Florida, United States; North Florida/South Georgia Veterans Health System, Gainesville, Florida, United States; VA Ann Arbor Healthcare System, Ann Arbor, Michigan, United States

## Abstract

**Background:**

Rural older Veterans face unique challenges in managing multiple chronic conditions. A novel, virtual Comprehensive Geriatric Assessment (CGA) clinic, at a southeastern Veterans Affairs (VA) medical center, aimed to optimize medications for this population. The objective of this study is to evaluate the geriatric clinical pharmacist (GCP) recommendations and their implementation for the CGA patients’ medication regimens.

**Methods:**

The study performed electronic health record review of North Florida/South Georgia VA CGA patients, focusing on GCP recommendations targeting potentially inappropriate medications (PIMs) and polypharmacy. Recommendations were categorized as: discontinue, switch, taper, request further provider review, address polypharmacy. Recommendations were evaluated as to whether they were implemented and when the implementation occurred.

**Results:**

The study population consisted of 273 Veterans. 86 Veterans received no recommendations. The GCP provided 323 recommendations for 187 Veterans, with 156 (48.3%) implemented. Recommendation uptake was highest for requesting provider review of a medication issue (58%); it was lowest for switching medications (27.9%). Implemented recommendations regarding medication categories included: non-anticholinergic BEERs Criteria medications, 36.3%; anticholinergic medications, 41.8%; hypoglycemic or hypotensive risk medications, 69%; general polypharmacy issues, 42.9%; other medications, 56.7%.

**Conclusion:**

This virtual GCP intervention, as part of a broader virtual CGA clinic, demonstrated successful implementation of deprescribing and medication optimizing recommendations, resulting in reduction in use of PIMs and polypharmacy in rural older rural Veterans. The intervention also resulted in increased collaboration with PCPs. Future evaluation needs to study the intervention’s long-term outcomes in decreasing polypharmacy and increasing medication safety for this vulnerable population.

